# (*E*)-2-Meth­oxy-*N*′-(4-nitro­benzyl­idene)benzohydrazide

**DOI:** 10.1107/S160053680905079X

**Published:** 2009-11-28

**Authors:** Hong-Yan Ban, Cong-Ming Li

**Affiliations:** a School of Chemical Engineering, University of Science and Technology Liaoning, Anshan 114051, People’s Republic of China; b College of Science, Shenyang University, Shenyang 110044, People’s Republic of China.

## Abstract

In the title compound, C_15_H_13_N_3_O_4_, the mol­ecule exists in a *trans* configuration with respect to the methyl­idene unit. The dihedral angle between the two benzene rings is 6.8 (2)°. The C—N—NH—C torsion angle is 3.4 (3)°. The mol­ecule possesses an intra­molecular N—H⋯O hydrogen bond. In the crystal structure, adjacent mol­ecules are linked through inter­molecular C—H⋯O hydrogen bonds, forming dimers

## Related literature

For the biological activity of hydrazones, see: Zhong *et al.* (2007 ▶); Raj *et al.* (2007 ▶); Jimenez-Pulido *et al.* (2008 ▶). For related structures, see: Ban & Li (2008*a* ▶,*b* ▶); Li & Ban (2009*a* ▶,*b* ▶); Yehye *et al.* (2008 ▶); Fun, Patil, Jebas *et al.* (2008 ▶); Fun, Patil, Rao *et al.* (2008 ▶); Yang *et al.* (2008 ▶); Ejsmont *et al.* (2008 ▶).
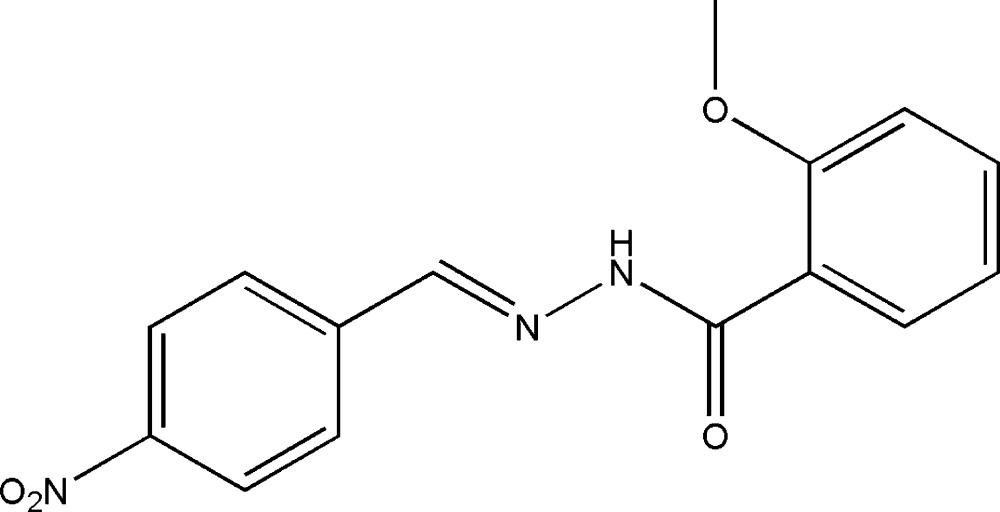


## Experimental

### 

#### Crystal data

C_15_H_13_N_3_O_4_
*M* *_r_* = 299.28
Monoclinic, 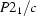

*a* = 11.1843 (2) Å
*b* = 11.3718 (3) Å
*c* = 13.0519 (2) Å
β = 121.792 (2)°
*V* = 1410.96 (6) Å^3^
*Z* = 4
Mo *K*α radiation
μ = 0.11 mm^−1^
*T* = 298 K
0.15 × 0.13 × 0.12 mm

#### Data collection

Bruker SMART CCD area-detector diffractometer
Absorption correction: multi-scan (*SADABS*; Sheldrick, 1996 ▶) *T*_min_ = 0.985, *T*_max_ = 0.988
8270 measured reflections
3048 independent reflections
1964 reflections with *I* > 2σ(*I*)
*R*_int_ = 0.026

#### Refinement

*R*[*F* ^2^ > 2σ(*F* ^2^)] = 0.047
*wR*(*F* ^2^) = 0.138
*S* = 1.02
3048 reflections
203 parameters
1 restraint
H atoms treated by a mixture of independent and constrained refinement
Δρ_max_ = 0.17 e Å^−3^
Δρ_min_ = −0.29 e Å^−3^

### 

Data collection: *SMART* (Bruker, 1998 ▶); cell refinement: *SAINT* (Bruker, 1998 ▶); data reduction: *SAINT*; program(s) used to solve structure: *SHELXS97* (Sheldrick, 2008 ▶); program(s) used to refine structure: *SHELXL97* (Sheldrick, 2008 ▶); molecular graphics: *SHELXTL* (Sheldrick, 2008 ▶); software used to prepare material for publication: *SHELXTL*.

## Supplementary Material

Crystal structure: contains datablocks global, I. DOI: 10.1107/S160053680905079X/wn2369sup1.cif

Structure factors: contains datablocks I. DOI: 10.1107/S160053680905079X/wn2369Isup2.hkl

Additional supplementary materials:  crystallographic information; 3D view; checkCIF report

## Figures and Tables

**Table 1:** Hydrogen-bond geometry (Å, °)

*D*—H⋯*A*	*D*—H	H⋯*A*	*D*⋯*A*	*D*—H⋯*A*
N2—H2*A*⋯O1	0.91 (1)	1.94 (2)	2.644 (2)	133 (2)
C3—H3⋯O2^i^	0.93	2.50	3.260 (2)	140

Symmetry code: (i) 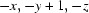.

**Fig. 1.**
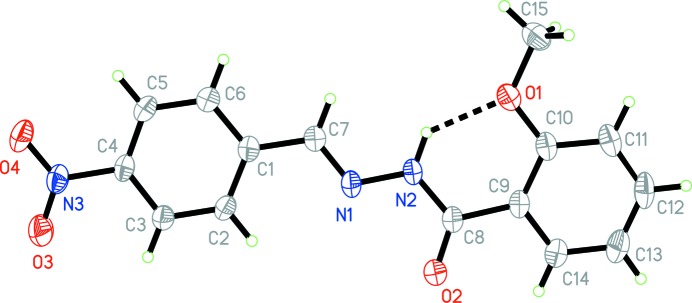
The molecular structure of the title compound, showing 30% probability displacement ellipsoids for the non-hydrogen atoms. Hydrogen atoms are shown as spheres of arbitrary radius. The intramolecular N—H···O hydrogen bond is shown as a dashed line.

**Fig. 2.**
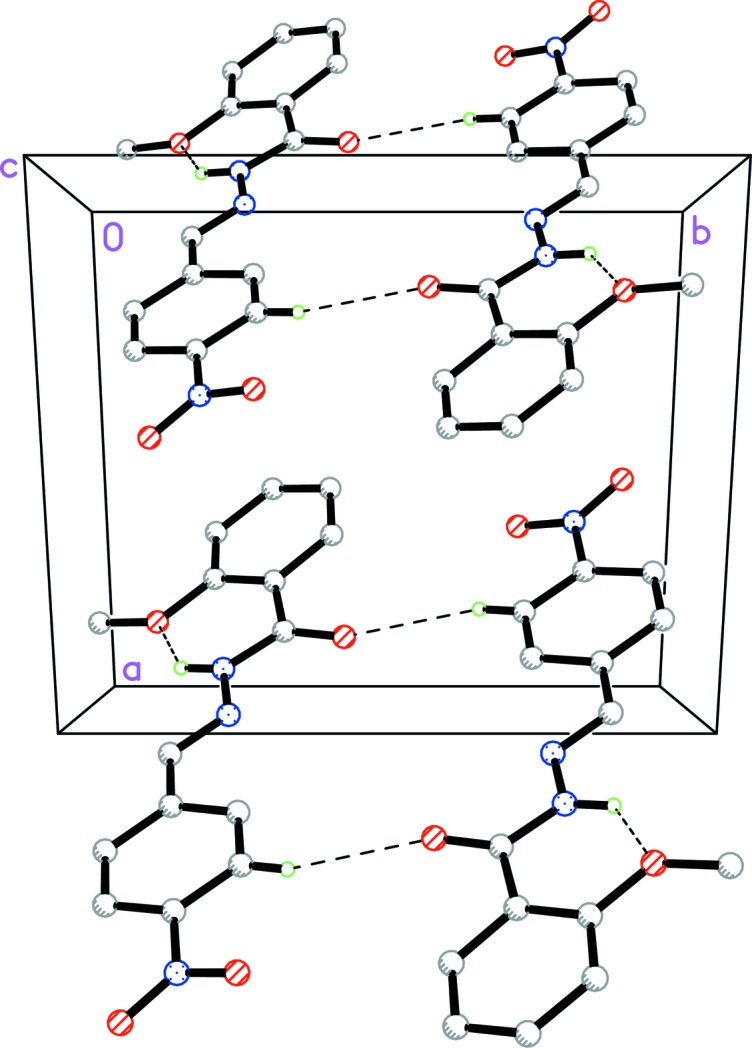
The molecular packing of the title compound, viewed along the c axis. Hydrogen bonds are shown as dashed lines. Hydrogen atoms not involved in these hydrogen bonds have been omitted.

## supplementary crystallographic information

### Comment

Hydrazones derived from the condensation of aldehydes with hydrazides have been shown to possess excellent biological activities (Zhong *et al.*, 2007; Raj *et al.*, 2007; Jimenez-Pulido *et al.*, 2008). Due to the easy synthesis of such compounds, a great deal of hydrazones have been synthesized and structurally characterized (Yehye *et al.*, 2008; Fun, Patil, Jebas *et al.*, 2008; Fun, Patil, Rao *et al.*, 2008; Yang *et al.*, 2008; Ejsmont *et al.*, 2008). Recently, we have also reported the crystal structures of a few hydrazones (Ban & Li, 2008a,b; Li & Ban, 2009a,b). In this paper, we report the crystal structure of the title compound.

In the structure of the title compound (Fig. 1) the molecule exists in a *trans* configuration with respect to the methylidene unit. The dihedral angle between the two benzene rings is 6.8 (2)°. In the 2-methoxyphenyl unit, the methoxy group is nearly coplanar with the mean plane of the C9–C14 ring; the atom C15 deviates from this plane by 0.002 (2) Å. The torsion angle C7—N1—N2—C8 is 3.4 (3)°. The molecule possesses an intramolecular N—H···O hydrogen bond (Table 1, Fig. 1).

In the crystal structure, adjacent molecules are linked through intermolecular C—H···O hydrogen bonds (Table 1), forming dimers (Fig. 2).

### Experimental

The compound was prepared by refluxing 4-nitrobenzaldehyde (1.0 mol) with 2-methoxybenzohydrazide (1.0 mol) in methanol (100 ml). Excess methanol was removed from the mixture by distillation. The colorless solid product was filtered, and washed three times with methanol. Colorless block crystals of the title compound were obtained from a methanol solution by slow evaporation in air.

### Refinement

H2A, attached to N2, was located in a difference Fourier map and refined isotropically, with the N—H distance restrained to 0.90 (1) Å. Other H atoms were placed in calculated positions (C—H = 0.93 - 0.96 Å) and refined as riding with U_iso_(H) = 1.5U_eq_(methyl C) and 1.2U_eq_(other C). A rotating group model was used for the methyl group.

### Crystal data

**Table:**

C_15_H_13_N_3_O_4_	*F*(000) = 624
*M**_r_* = 299.28	*D*_x_ = 1.409 Mg m^−^^3^
Monoclinic, *P*2_1_/*c*	Mo *K*α radiation, λ = 0.71073 Å
Hall symbol: -P 2ybc	Cell parameters from 2137 reflections
*a* = 11.1843 (2) Å	θ = 2.6–27.7°
*b* = 11.3718 (3) Å	µ = 0.11 mm^−^^1^
*c* = 13.0519 (2) Å	*T* = 298 K
β = 121.792 (2)°	Block, colorless
*V* = 1410.96 (6) Å^3^	0.15 × 0.13 × 0.12 mm
*Z* = 4	

### Data collection

**Table:**

Bruker SMART CCD area-detector diffractometer	3048 independent reflections
Radiation source: fine-focus sealed tube	1964 reflections with *I* > 2σ(*I*)
graphite	*R*_int_ = 0.026
ω scans	θ_max_ = 27.0°, θ_min_ = 2.1°
Absorption correction: multi-scan (*SADABS*; Sheldrick, 1996)	*h* = −14→13
*T*_min_ = 0.985, *T*_max_ = 0.988	*k* = −14→9
8270 measured reflections	*l* = −16→15

### Refinement

**Table:**

Refinement on *F*^2^	Primary atom site location: structure-invariant direct methods
Least-squares matrix: full	Secondary atom site location: difference Fourier map
*R*[*F*^2^ > 2σ(*F*^2^)] = 0.047	Hydrogen site location: inferred from neighbouring sites
*wR*(*F*^2^) = 0.138	H atoms treated by a mixture of independent and constrained refinement
*S* = 1.02	*w* = 1/[σ^2^(*F*_o_^2^) + (0.0691*P*)^2^ + 0.1341*P*] where *P* = (*F*_o_^2^ + 2*F*_c_^2^)/3
3048 reflections	(Δ/σ)_max_ < 0.001
203 parameters	Δρ_max_ = 0.17 e Å^−^^3^
1 restraint	Δρ_min_ = −0.29 e Å^−^^3^

### Special details

**Table:**

Geometry. All esds (except the esd in the dihedral angle between two l.s. planes) are estimated using the full covariance matrix. The cell esds are taken into account individually in the estimation of esds in distances, angles and torsion angles; correlations between esds in cell parameters are only used when they are defined by crystal symmetry. An approximate (isotropic) treatment of cell esds is used for estimating esds involving l.s. planes.
Refinement. Refinement of F^2^ against ALL reflections. The weighted R-factor wR and goodness of fit S are based on F^2^, conventional R-factors R are based on F, with F set to zero for negative F^2^. The threshold expression of F^2^ > 2sigma(F^2^) is used only for calculating R-factors(gt) etc. and is not relevant to the choice of reflections for refinement. R-factors based on F^2^ are statistically about twice as large as those based on F, and R- factors based on ALL data will be even larger.

### Fractional atomic coordinates and isotropic or equivalent isotropic displacement parameters (Å^2^)

**Table:**

	*x*	*y*	*z*	*U*_iso_*/*U*_eq_	
N1	0.02468 (14)	0.74628 (13)	0.11097 (11)	0.0533 (4)	
N2	0.10276 (15)	0.75892 (13)	0.23349 (11)	0.0542 (4)	
N3	−0.37828 (15)	0.80034 (14)	−0.46112 (12)	0.0584 (4)	
O1	0.18128 (13)	0.87506 (11)	0.43486 (10)	0.0632 (4)	
O2	0.16350 (19)	0.56837 (12)	0.25823 (12)	0.0989 (6)	
O3	−0.36814 (16)	0.71210 (14)	−0.50895 (11)	0.0841 (5)	
O4	−0.45977 (16)	0.87945 (13)	−0.51687 (11)	0.0874 (5)	
C1	−0.12755 (16)	0.82699 (14)	−0.08248 (13)	0.0477 (4)	
C2	−0.13227 (17)	0.72335 (14)	−0.14102 (14)	0.0513 (4)	
H2	−0.0804	0.6587	−0.0958	0.062*	
C3	−0.21222 (17)	0.71486 (15)	−0.26456 (14)	0.0518 (4)	
H3	−0.2148	0.6454	−0.3032	0.062*	
C4	−0.28832 (16)	0.81139 (14)	−0.32966 (13)	0.0477 (4)	
C5	−0.28638 (18)	0.91571 (15)	−0.27587 (15)	0.0572 (4)	
H5	−0.3389	0.9798	−0.3219	0.069*	
C6	−0.20458 (18)	0.92326 (15)	−0.15175 (15)	0.0559 (4)	
H6	−0.2009	0.9936	−0.1139	0.067*	
C7	−0.04485 (17)	0.83471 (15)	0.04892 (14)	0.0523 (4)	
H7	−0.0432	0.9044	0.0870	0.063*	
C8	0.17233 (18)	0.66437 (16)	0.30264 (14)	0.0571 (4)	
C9	0.26280 (16)	0.68339 (15)	0.43602 (13)	0.0507 (4)	
C10	0.26528 (17)	0.78298 (15)	0.49941 (13)	0.0514 (4)	
C11	0.35310 (19)	0.78490 (18)	0.62478 (15)	0.0643 (5)	
H11	0.3528	0.8500	0.6678	0.077*	
C12	0.4398 (2)	0.6917 (2)	0.68511 (16)	0.0730 (6)	
H12	0.4982	0.6944	0.7686	0.088*	
C13	0.4412 (2)	0.5949 (2)	0.62399 (16)	0.0720 (6)	
H13	0.5015	0.5326	0.6653	0.086*	
C14	0.35239 (19)	0.59045 (17)	0.50043 (15)	0.0621 (5)	
H14	0.3523	0.5238	0.4591	0.075*	
C15	0.1754 (2)	0.97515 (17)	0.49824 (18)	0.0720 (6)	
H15A	0.1474	0.9507	0.5530	0.108*	
H15B	0.1083	1.0306	0.4417	0.108*	
H15C	0.2665	1.0114	0.5426	0.108*	
H2A	0.102 (2)	0.8277 (12)	0.2684 (17)	0.080*	

### Atomic displacement parameters (Å^2^)

**Table:**

	*U*^11^	*U*^22^	*U*^33^	*U*^12^	*U*^13^	*U*^23^
N1	0.0562 (8)	0.0618 (9)	0.0314 (7)	0.0037 (7)	0.0159 (6)	−0.0002 (6)
N2	0.0613 (9)	0.0581 (9)	0.0295 (7)	0.0065 (7)	0.0145 (6)	−0.0012 (6)
N3	0.0601 (9)	0.0703 (10)	0.0373 (7)	0.0068 (8)	0.0204 (7)	0.0109 (7)
O1	0.0706 (8)	0.0654 (8)	0.0440 (6)	0.0069 (6)	0.0237 (6)	−0.0082 (6)
O2	0.1488 (15)	0.0625 (9)	0.0423 (7)	0.0271 (9)	0.0207 (8)	−0.0018 (6)
O3	0.1009 (11)	0.0921 (11)	0.0426 (7)	0.0188 (8)	0.0264 (7)	−0.0010 (7)
O4	0.0927 (11)	0.0905 (10)	0.0455 (7)	0.0317 (8)	0.0135 (7)	0.0184 (7)
C1	0.0465 (9)	0.0548 (10)	0.0384 (8)	0.0013 (7)	0.0200 (7)	0.0037 (7)
C2	0.0539 (9)	0.0515 (10)	0.0405 (8)	0.0102 (7)	0.0195 (7)	0.0098 (7)
C3	0.0561 (10)	0.0537 (10)	0.0395 (8)	0.0074 (7)	0.0209 (7)	0.0023 (7)
C4	0.0454 (8)	0.0590 (10)	0.0342 (8)	0.0042 (7)	0.0178 (7)	0.0091 (7)
C5	0.0594 (10)	0.0542 (10)	0.0471 (9)	0.0126 (8)	0.0204 (8)	0.0150 (8)
C6	0.0601 (10)	0.0521 (10)	0.0456 (9)	0.0072 (8)	0.0210 (8)	0.0021 (7)
C7	0.0546 (10)	0.0542 (10)	0.0400 (8)	0.0029 (8)	0.0194 (7)	−0.0003 (7)
C8	0.0660 (11)	0.0585 (11)	0.0359 (8)	0.0066 (8)	0.0193 (8)	0.0009 (8)
C9	0.0494 (9)	0.0630 (11)	0.0341 (8)	−0.0003 (8)	0.0183 (7)	0.0033 (7)
C10	0.0471 (9)	0.0657 (11)	0.0367 (8)	−0.0049 (8)	0.0189 (7)	−0.0020 (7)
C11	0.0622 (11)	0.0859 (14)	0.0389 (9)	−0.0129 (10)	0.0225 (8)	−0.0119 (9)
C12	0.0604 (11)	0.1071 (17)	0.0325 (9)	−0.0109 (11)	0.0115 (8)	0.0056 (10)
C13	0.0623 (12)	0.0882 (15)	0.0474 (10)	0.0045 (10)	0.0165 (9)	0.0172 (10)
C14	0.0630 (11)	0.0681 (12)	0.0453 (9)	0.0049 (9)	0.0217 (8)	0.0073 (8)
C15	0.0915 (14)	0.0618 (12)	0.0640 (12)	−0.0047 (10)	0.0418 (11)	−0.0136 (9)

### Geometric parameters (Å, °)

**Table:**

N1—C7	1.268 (2)	C5—C6	1.381 (2)
N1—N2	1.3672 (17)	C5—H5	0.9300
N2—C8	1.355 (2)	C6—H6	0.9300
N2—H2A	0.908 (9)	C7—H7	0.9300
N3—O4	1.2127 (18)	C8—C9	1.498 (2)
N3—O3	1.2183 (18)	C9—C14	1.393 (2)
N3—C4	1.467 (2)	C9—C10	1.394 (2)
O1—C10	1.3619 (19)	C10—C11	1.396 (2)
O1—C15	1.429 (2)	C11—C12	1.371 (3)
O2—C8	1.215 (2)	C11—H11	0.9300
C1—C2	1.390 (2)	C12—C13	1.364 (3)
C1—C6	1.392 (2)	C12—H12	0.9300
C1—C7	1.460 (2)	C13—C14	1.379 (2)
C2—C3	1.375 (2)	C13—H13	0.9300
C2—H2	0.9300	C14—H14	0.9300
C3—C4	1.375 (2)	C15—H15A	0.9600
C3—H3	0.9300	C15—H15B	0.9600
C4—C5	1.373 (2)	C15—H15C	0.9600
			
C7—N1—N2	117.55 (14)	C1—C7—H7	120.1
C8—N2—N1	118.97 (14)	O2—C8—N2	121.41 (15)
C8—N2—H2A	120.2 (13)	O2—C8—C9	121.28 (15)
N1—N2—H2A	120.6 (13)	N2—C8—C9	117.30 (15)
O4—N3—O3	123.15 (15)	C14—C9—C10	118.37 (14)
O4—N3—C4	118.28 (15)	C14—C9—C8	114.99 (15)
O3—N3—C4	118.56 (14)	C10—C9—C8	126.64 (15)
C10—O1—C15	118.79 (13)	O1—C10—C9	117.77 (13)
C2—C1—C6	118.59 (14)	O1—C10—C11	122.89 (16)
C2—C1—C7	120.89 (14)	C9—C10—C11	119.34 (16)
C6—C1—C7	120.52 (15)	C12—C11—C10	120.60 (18)
C3—C2—C1	121.13 (14)	C12—C11—H11	119.7
C3—C2—H2	119.4	C10—C11—H11	119.7
C1—C2—H2	119.4	C13—C12—C11	120.70 (16)
C4—C3—C2	118.51 (15)	C13—C12—H12	119.7
C4—C3—H3	120.7	C11—C12—H12	119.7
C2—C3—H3	120.7	C12—C13—C14	119.37 (18)
C5—C4—C3	122.42 (14)	C12—C13—H13	120.3
C5—C4—N3	119.23 (14)	C14—C13—H13	120.3
C3—C4—N3	118.31 (15)	C13—C14—C9	121.58 (18)
C4—C5—C6	118.42 (15)	C13—C14—H14	119.2
C4—C5—H5	120.8	C9—C14—H14	119.2
C6—C5—H5	120.8	O1—C15—H15A	109.5
C5—C6—C1	120.92 (16)	O1—C15—H15B	109.5
C5—C6—H6	119.5	H15A—C15—H15B	109.5
C1—C6—H6	119.5	O1—C15—H15C	109.5
N1—C7—C1	119.88 (16)	H15A—C15—H15C	109.5
N1—C7—H7	120.1	H15B—C15—H15C	109.5

### Hydrogen-bond geometry (Å, °)

**Table:**

*D*—H···*A*	*D*—H	H···*A*	*D*···*A*	*D*—H···*A*
N2—H2A···O1	0.91 (1)	1.94 (2)	2.644 (2)	133 (2)
C3—H3···O2^i^	0.93	2.50	3.260 (2)	140

Symmetry codes: (i) −*x*, −*y*+1, −*z*.
